# Supplementary material for: Effects of intraspecies and interspecies competition on genetic device construction and performance
Source: mSphere. 2025 Dec 17;11(1):e00402-25. doi: 10.1128/msphere.00402-25 (PMC12838364; doi:10.1128/msphere.00402-25)
Supplement: Supplemental material — Figures S1 to S3 and Tables S1 to S3. [file msphere.00402-25-s0001.docx]

**
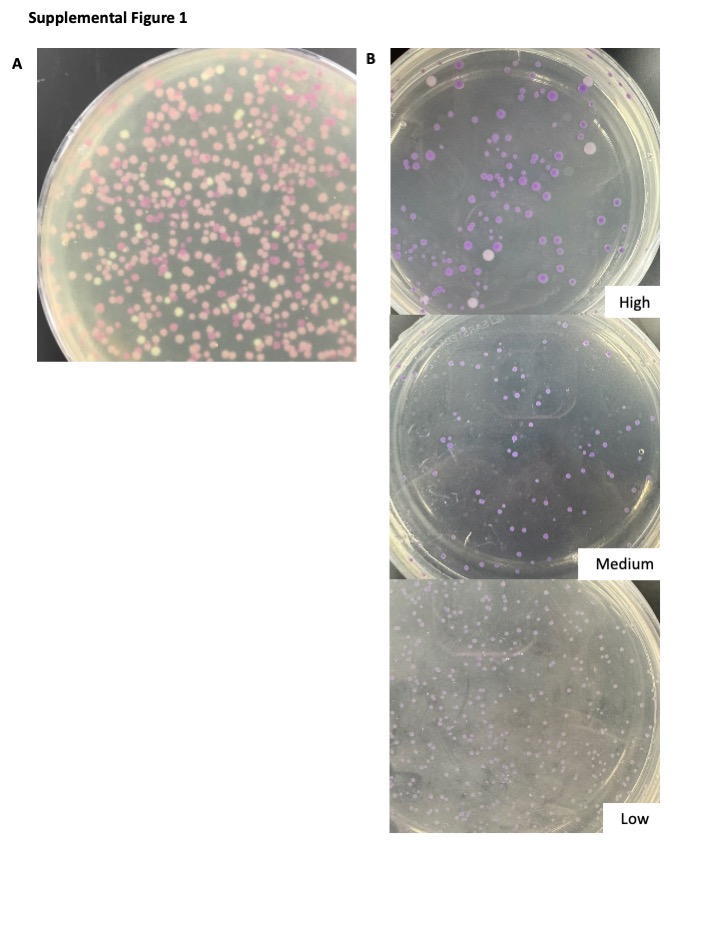
**

**Supplemental Figure 1.** *Examples of highly variable chromoprotein expression.* **A)** Spread plate of high strength promoter driving Cupid Pink chromoprotein expression from the pUC19 plasmid in *E. coli* DH5alpha cells grown overnight in LB media under antibiotic selection. **B)** Spread plates of J23100 (high, top), J23108 (medium, center), and J23110 (low, bottom) promoters driving tsP chromoprotein expression from the pSB1C3 plasmid.


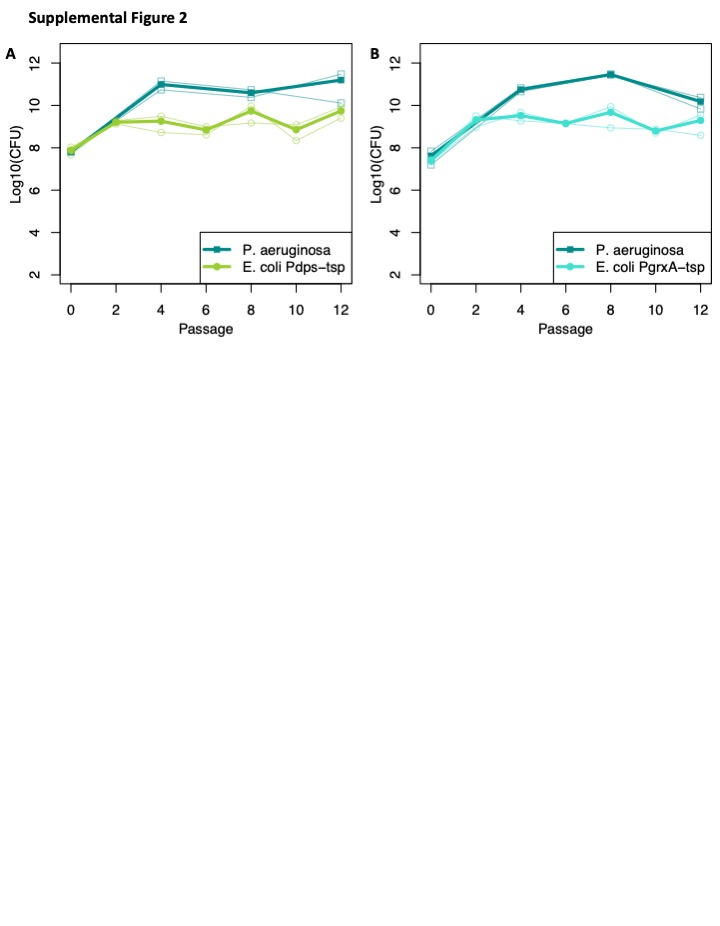


**Supplemental Figure 2.** *Regulated promoter genetic devices do not alter the proportion of* E. coli *and* P. aeruginosa *in co-culture.* **A)** Colony counts for *E. coli* expressing *P_dps_-tsP* (green) co-cultured with *P. aeruginosa* (dark blue). N = 4. **B)** Colony counts for *E. coli* expressing *P_grxA_-tsP* (blue) co-cultured with *P. aeruginosa* (dark blue). N = 4. Thin lines show individual experiments, thick lines show the average. Student’s paired t-test were conducted comparing *E. coli* containing each genetic device with *E. coli* (*P_dps_-tsP* = 0.410, *P_grxA_-tsP* = 0.499) and the *P. aeruginos*a from co-culture with *E. coli* containing the genetic device with *P. aeruginos*a from co-culture with *E. coli* (*P_dps_-tsP* = 0.054, *P_grxA_-tsP* = 0.168).


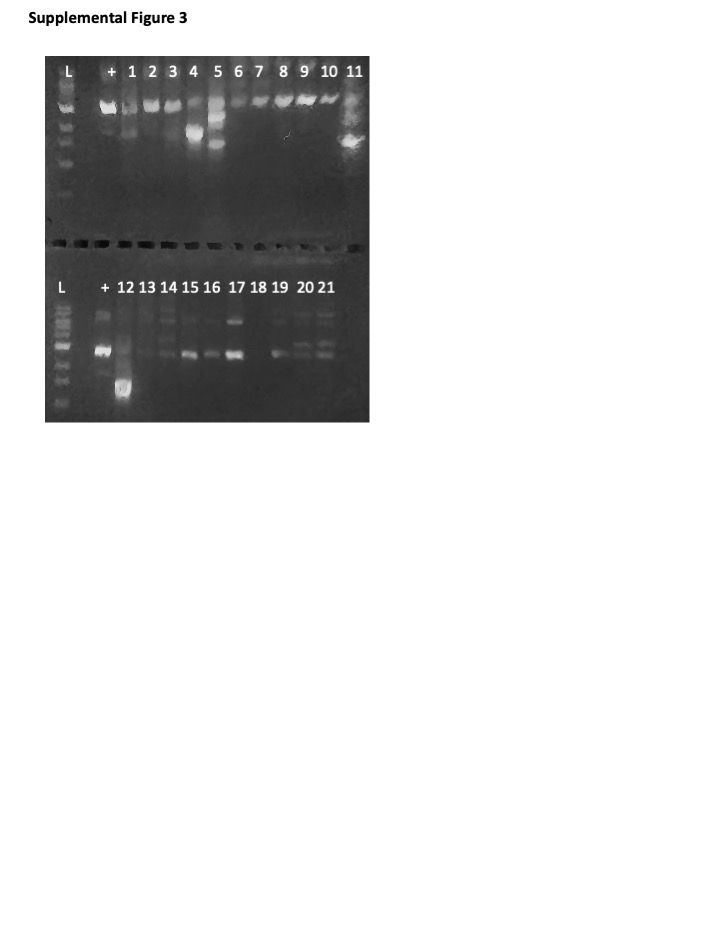


**Supplemental Figure 3.** *Diagnostic PCR shows the plasmid containing the P_dps_-tsP device is present in the wastewater culture.* + = tsPurple plasmid positive control, L = 1kb DNA ladder. PCR of plasmids isolated from cell pellets shown in Figure 6B. Lanes 1 – 7 = Biological replicate 1, Lanes 8 – 14 = Biological replicate 2, Lanes 15 – 21 = Biological replicate 3.


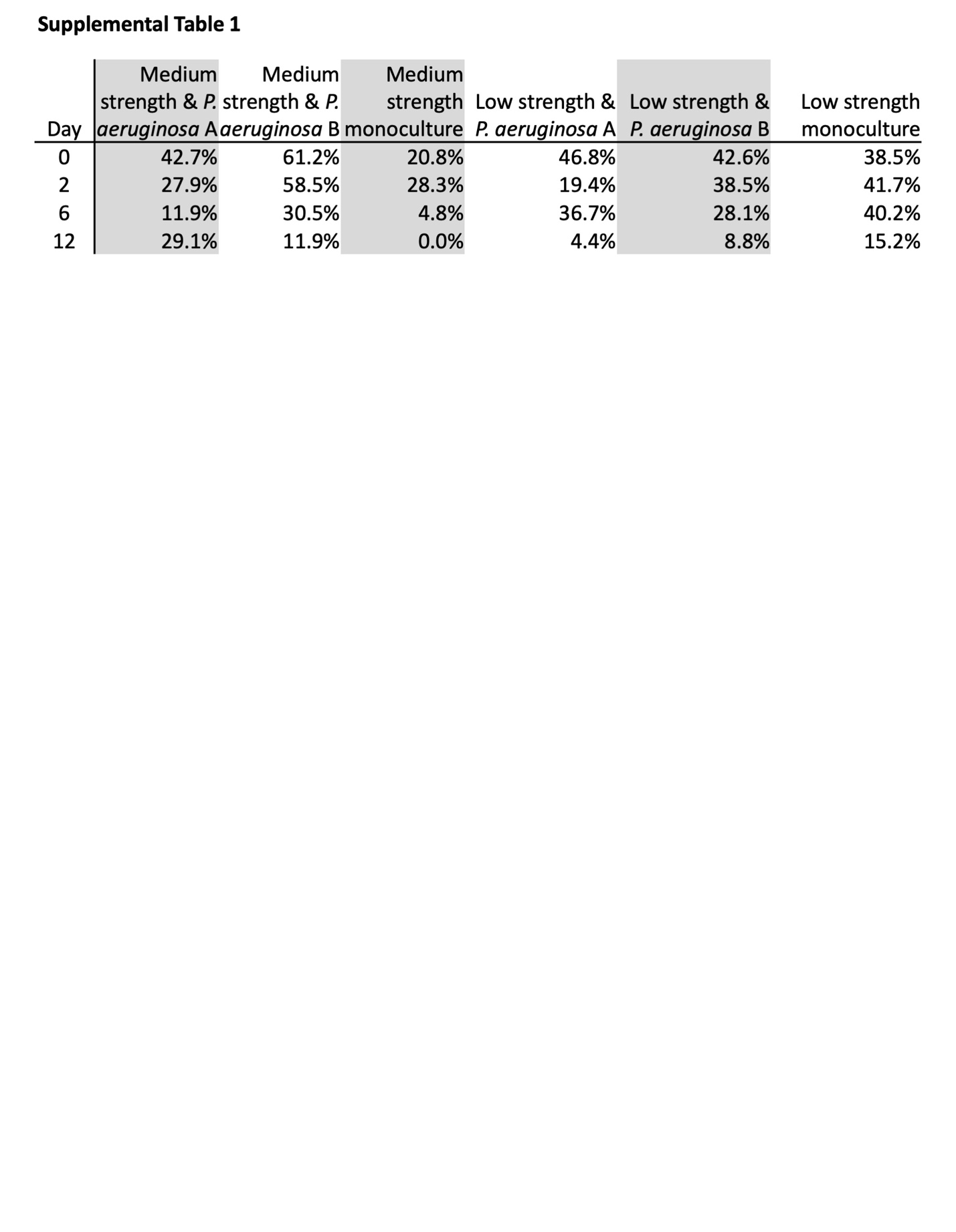


**Supplemental Table 1.** *Removal of antibiotic selection results in loss of tsP expression.* Percentage of *E. coli* colonies for medium and low strength Anderson promoters expressing tsP in co-culture with *P. aeruginosa* is similar to monoculture when antibiotic selection to maintain the plasmid is removed. Each column is a biological replicate.


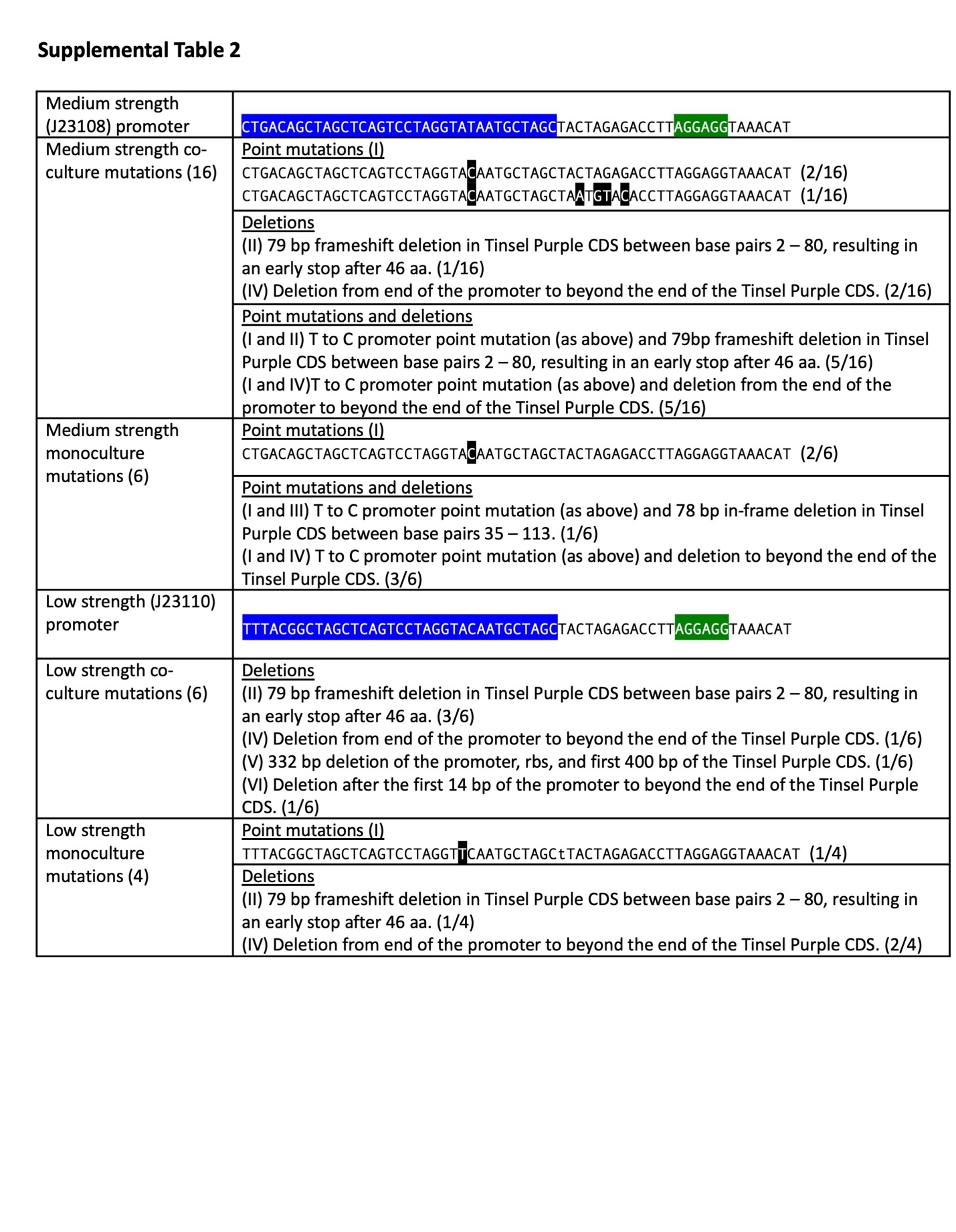


**Supplemental Table 2.** Different mutations from Figure 3G organized by Anderson promoter used and whether the *E. coli* with the specific genetic device was grown in co-culture with *P. aeruginosa* PAO1 or in monoculture. Blue highlighted text = promoter. Green highlighted text = RBS. Black highlighted text = point mutations. Numbers in parenthesis = (frequency of mutation/total mutations analyzed)


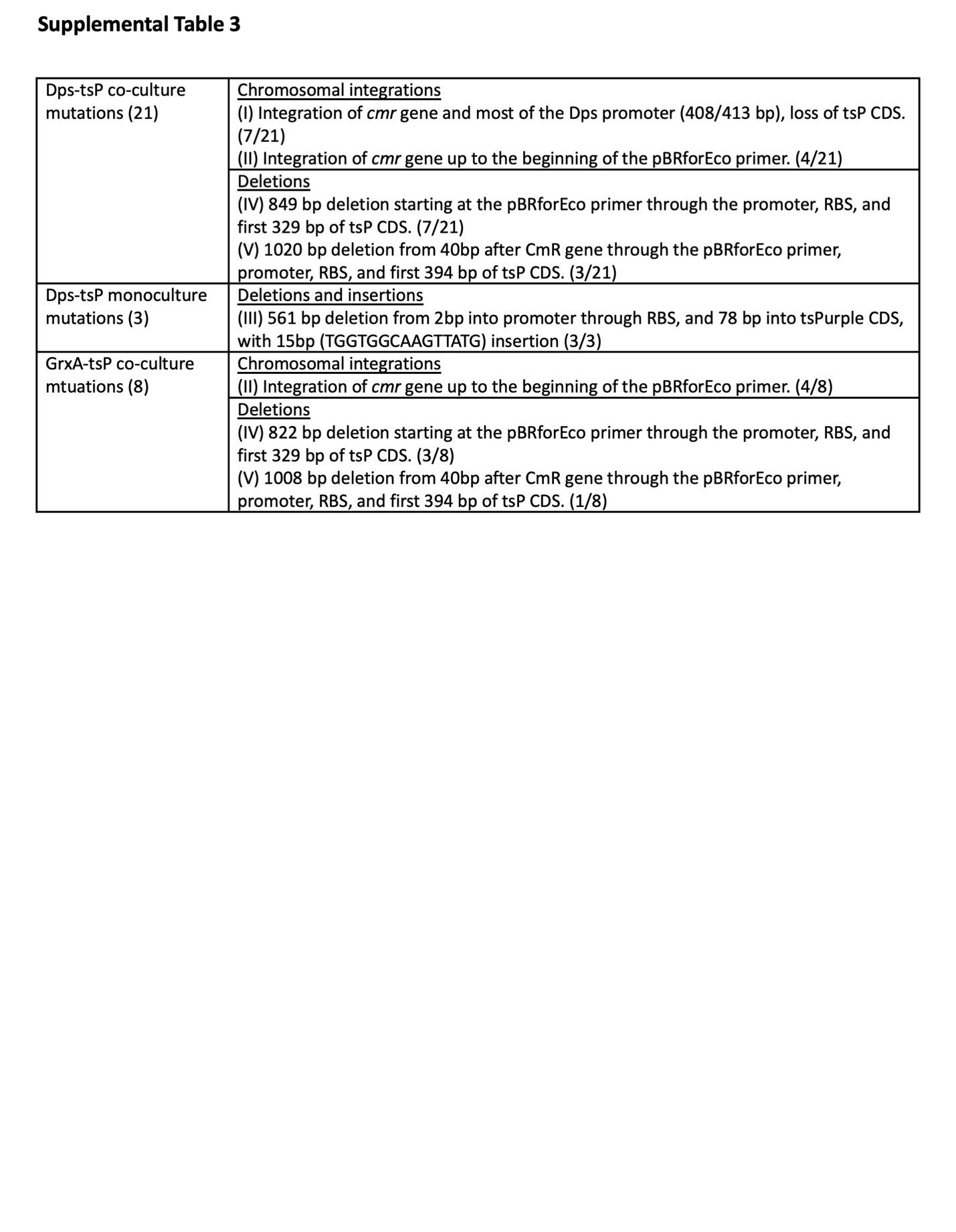


**Supplemental Table 3.** Different mutations from Figure 5C organized by genetic device and whether the *E. coli* with the genetic device was grown in co-culture with *P. aeruginosa* or in monoculture. Numbers in parenthesis = (frequency of mutation/total mutations analyzed)
